# Diet in Early Life Is Related to Child Mental Health and Personality at 8 Years: Findings from the Norwegian Mother, Father and Child Cohort Study (MoBa)

**DOI:** 10.3390/nu15010243

**Published:** 2023-01-03

**Authors:** Kristine Vejrup, Elisabet R. Hillesund, Neha Agnihotri, Christine Helle, Nina C. Øverby

**Affiliations:** 1 Norwegian Armed Forces Joint Medical Services, Institute of Military Epidemiology, 0450 Oslo, Norway; 2 Department of Nutrition and Public Health, University of Agder, 4630 Kristiansand, Norway

**Keywords:** MoBa, the Norwegian Mother, Father and Child Cohort Study, NND score, child behaviour and personality types, MBRN

## Abstract

There is rising concern about population mental health. Personality and mental health traits manifest early. Sufficient nutrition is fundamental to early development. However, little is known about early life dietary impact on later mental health. The aim of this study was to investigate associations of exposure to a healthy and sustainable antenatal and early childhood diet with personality traits and symptoms of depression and anxiety measured at 8 years of age. This study is based on the Norwegian Mother, Father and Child Cohort Study (MoBa) and uses data from the Medical Birth Registry of Norway (MBRN) including 40,566 participants. Mental health measures and personality traits were assessed at 8 years. Dietary data from pregnancy, child age 6 and 18 months and 3 and 7 years were used. With few exceptions, inverse associations were observed between healthier diet at all time points and depression and anxiety symptom scores at age 8. We found positive associations between diet scores at almost all time points and extraversion, benevolence, conscientiousness and imagination. Inverse associations were observed between diet scores and neuroticism. Combined, these findings underpin a probable impact of both maternal pregnancy diet and early childhood diet on several aspects of child mental health.

## 1. Introduction

The period in a child’s life from conception until 2 years of age is a crucial growth and development phase, where all organs, regulatory systems and physiology are set in place, and also set foundations for mental health, personality and socio-emotional development [1,2]. The food and nurture provided in this phase are at the core of this development. Adequate nutrition, such as breastmilk or formula, and later nutritious and healthy complementary foods, are necessary for normal physiological development, including brain development [3,4,5]. Iodine, iron and long chain fatty acids are particularly important for the normal development of the brain, and shortage of these nutrients in critical phases may cause irreversible harm and cognitive and motoric delay [6,7]. The actual feeding events or meals are by themselves important events for interaction and nurture involved in socio-emotional development [8]. The child mimics caregivers and family and acquires skills that are important throughout life [9]. Pregnancy and the first years of life are therefore a crucial time for optimal nourishment.

There is rising concern about population mental health and wellbeing [10]. Despite policies aimed at reducing mental health disorders, not much has changed in the past two decades at the global level [10]. Relative to other non-communicable diseases depression and anxiety manifest at an early age, with life-long loss of health (DALYs) observed as early as 5–9 years. Prevalence of depression and anxiety increase across childhood and adolescence [10]. This early origin of mental disability calls for prevention and identification of modifiable determinants of mental health.

Compromised early-life nutritional health could influence individual vulnerability to mental health problems. Associations of maternal diet with measures of offspring mental health and Attention Deficit Hyperactivity Disorder (ADHD) have been investigated in the Mother, Father and Child Cohort study (MoBa) by Jacka et al. (2013) and Borge et al. (2021), respectively [11,12]. Both report observations of independent associations between antenatal nutritional exposures and aspects of mental health, including behavioral and emotional problems in children [11] and risk of ADHD [12], respectively. Others have reported associations between maternal diet and offspring neurodevelopment [13,14], suggesting that healthier diet is associated with better neurodevelopmental outcomes. There is a lack of studies with long term follow up, such as up to school age [3,12]. A few studies have also explored the relation between early childhood diet and later mental health status [11,12]. Jacka et al. found that lower intake of healthy foods in early childhood was associated with higher levels of both internalizing and externalizing problems, while Borge et al. (2021) found no association with early postnatal diet quality and ADHD [12].

Personality traits are often presented in five different, but somewhat correlated traits: extraversion, benevolence, conscientiousness, neuroticism, and imagination, also commonly referred to as the Big Five [15]. According to Steel et al., the five personality factors combined account for 39–63% of the variance in emotional well-being [16,17] and is also related to mental health [18,19]. Still, to the best of our knowledge, no studies have addressed longitudinal associations between early life diet and personality traits later in life. However, Schoeps et al. [18] and House et al. [20] have demonstrated associations between a healthy maternal diet and favorable child temperament. In the current study we expand this field by investigating on potential associations between maternal and child diet and personality traits as assessed by the Big Five.

We have previously developed a New Nordic Diet (NND) score assessing adherence to a healthy and a potentially sustainable dietary pattern, acknowledging the impact of diet on sustainability issues [21,22]. Maternal and child adherence to this healthy and potentially sustainable dietary pattern in the MoBa cohort was positively associated with measures of cognitive development up to five years of age as well as reduced risk of cognitive developmental delay [23]. The maternal NND score has also been shown to be positively associated with birthweight and protectively associated with preeclampsia and preterm delivery risk in MoBa [21], i.e., conditions that may impact child development [24,25].

The aim of the present study was to investigate potential associations of exposure to a healthy and sustainable antenatal and early childhood diet with personality traits and child symptoms of depression and anxiety as reported by parents at 8 years of age in the MoBa cohort.

## 2. Materials and Methods

### 2.1. Data Sources

The study was based on data from the Norwegian Mother, Father and Child Cohort Study (MoBa) and the Medical Birth Registry of Norway (MBRN) [26,27]. MoBa is a prospective population-based pregnancy cohort study conducted by the Norwegian Institute of Public Health (NIPH). Participants were recruited from all over Norway from 1999 to 2008. The women consented to participation in 41% of the pregnancies. The cohort includes approximately 114,500 children, 95,200 mothers and 75,200 fathers. The current study is based on version 12 of the quality-assured data files released for research in 2019. All questionnaires are available at the website of NIPH https://www.fhi.no/en/studies/moba/for-forskere-artikler/questionnaires-from-moba/ (accessed on 12 December 2022) [28].

### 2.2. Study Population Inclusion and Exclusion Criteria

**Figure 1:**
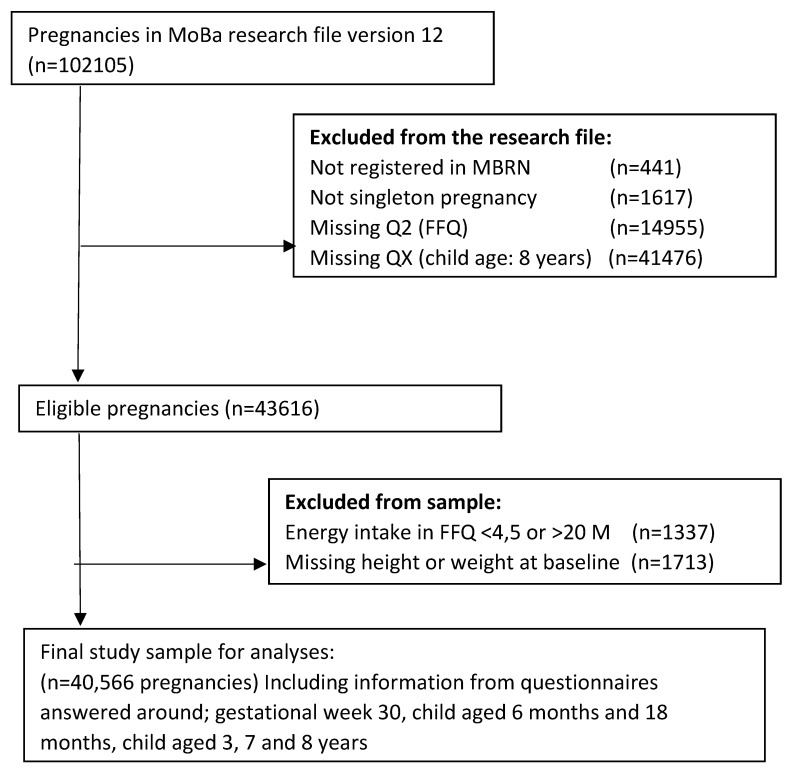
Flow chart inclusion of participants in the study.

The study population included participants who had responded to the baseline questionnaire around gestational week 17, the food frequency questionnaire (FFQ) [29] answered around gestational week 22 and were registered in the MBRN with singleton births. Criteria for inclusion in the present analyses was that the mother had answered the questionnaire distributed when the child was 8 years. We excluded women who did not report height or weight at baseline and those with calculated energy intakes outside the range 4.5–20 MJ/day. The final study population consisted of 40,566 mother–child pairs, including information from questionnaires answered at child aged 6 months and 18 months, child aged 3, 7 and 8 years (Figure 1).

### 2.3. Exposure

NND scores for maternal pregnancy diet and child diet at 6 and 18 months and 3 and 7 years have been developed and are described in detail in previously published papers [21,22,30]. In short, the rationale of the maternal score was to include food items and dietary habits considered to be both healthy and possibly sustainable, focusing on local foods in Nordic countries [21]. Based on this, a score comprising 10 subscales was developed. The subscales of the maternal New Nordic Diet (NND) score in MoBa are presented here (Table S1).

The child NND scores were developed under the rationale of being as similar as possible to the maternal NND score [21]. Despite referring to the child diet scores as child NND scores in this paper for simplicity reasons, it should be noted that they may not reflect the NND to the same extent as the maternal score. This is due to the questions assessing child diet being far less extensive and specific compared to the maternal FFQ, resulting in child scores that could only partially capture the intended aspects of the NND. For each child score, dietary variables from the child questionnaires were selected to construct subscales based on the subscales within the maternal NND score. The score at 6 months was specifically adapted to include subscales assessing breastfeeding and homemade foods as part of a healthy and potentially sustainable infant diet. The sum of the subscales was further computed into continuous age-specific NND scores and each score was divided into low, medium, and high adherence groups with the intention to create as equally sized groups as possible. The rationale for categorizing participants into low, medium, and high adherence was to compare associations to high vs. lower adherence to the described dietary pattern.

### 2.4. Outcomes

The main outcomes in this study were child symptoms of depression and anxiety as well as personality traits reported with questionnaire data by mothers at child aged 8 years. Key items in the questionnaire had been selected from standardized, validated scales widely used for assessing child behaviour and personality into shortened scale versions for the MoBa cohort [31,32,33,34,35,36,37,38,39] (Detailed information is available in Table S2). Two screening tools were used to investigate child anxiety and depression symptoms.

### 2.5. Anxiety Symptoms

The Screen for child anxiety-related disorders (SCARED) [31] is a multidimensional questionnaire that purports to measure anxiety symptoms in children and adolescents. The 5-item short version, as used in the MoBa, was developed in Birmaher et al. (1999), and has showed similar psychometrics to the full scale [32]. The scale has good internal consistency, assessed by means of Cronbach’s Alpha (0.70–0.90), as well as good test–retest reliability (*p* = 0.6–0.9). It has shown good discriminant validity in differentiating between youths with and without anxiety disorders and is a valid screening instrument to rate anxiety symptoms of children and adolescents. Mothers rate how true the statements describe their children using a 3-point scale (i.e., 1 = Not true, 2 = Sometimes true, 3 = True). A higher score reports more anxiety symptoms in the child.

### 2.6. Depression Symptoms

The Short Mood and Feelings Questionnaire (SMFQ) is a brief measure of childhood and adolescent depression, designed for rapid evaluation of core depressive symptomatology or for use in epidemiological studies. The original screening tool, the Mood and Feelings Questionnaire (MFQ) [33], is a 32-item questionnaire created to measure depression. The MFQ consists of a series of descriptive phrases regarding how the subject has been feeling or acting recently. A 13-item short form was developed, based on the discriminating ability between the depressed and non-depressed [34]. The parent version is used in the MoBa 8-year questionnaire. Its scaling properties as a potential dimensional measure of symptom severity of childhood depression was confirmed in community samples [35]. Mothers rate how true the statements describe their children using a 3-point scale (i.e., 1 = Not true, 2 = Sometimes true, 3 = True). A higher score reports more depression symptoms in the child.

### 2.7. Personality Traits

Child personality factors was measured with the short form of the Hierarchical Personality Inventory for Children (NHiPIC-30). NHiPIC is a reliable and valid measure of Norwegian children’s Big Five personality domains [36]. The original scale of The Hierarchical Personality Inventory for Children (HiPIC) [37,38] is a questionnaire with 144-items measuring the Big Five personality factors in children and adolescents. The short form consists of 30-items, also referred to as NHiPIC-30 [36]. It contains five broad personality traits domain scales with 6 items each: Extraversion, Benevolence, Conscientiousness, Neuroticism, Imagination (Table S2). Both the full and the short scale have been validated in Norwegian samples. For the Norwegian translation of the HiPIC full scale (NHiPIC), Cronbach’s alphas for the broad trait scales were 0.90 for extraversion, 0.98 for benevolence, 0.87 for conscientiousness, 0.86 for neuroticism, and 0.86 for imagination [39]. The NHiPIC reproduced five reliable and valid factors with excellent correspondence to the original measure, a hierarchical structure similar to that found for other Big Five assessment instruments for children. The short form (NHiPIC-30) correlated 0.90 with its longer counterpart [36]. Mothers rate how true the statements describe their child using a 5-point scale ranging from ‘not typical’ (1) to very typical (5). In the analysis, we have changed the score direction on some of the sub-questions so that behaviours that are not typical for the personality type have a low score and behaviours that are typical for the personality type ha a high score.

### 2.8. Potential Confounders

The following potential confounders were chosen prior to the analyses, due to their relevance for exposure and outcome. Information about maternal age (years) at delivery, parity and child sex was obtained from the MBRN. From the first MoBa questionnaire we obtained information about maternal education level (number of years school attendance: ≤12, 13–16, ≥17 years or missing), marital status (cohabiting or not). Maternal energy intake (KCAL) was obtained from the MoBa FFQ. Furthermore, from the first MoBa questionnaire we also obtained information on maternal depression symptoms based on a short version of the Hopkins Symptom Checklist, and information about pre-pregnancy maternal weight and height for calculating BMI. BMI was categorized according to the WHO classification as underweight (<18.5 kg/m^2^), normal weight (18.5–24.9 kg/m^2^), overweight (25.0–29.9 kg/m^2^) or obese (≥30.0 kg/m^2^).

### 2.9. Ethics

The establishment of MoBa and initial data collection was based on a license from the Norwegian Data Protection Agency and approval from The Regional Committees for Medical and Health Research Ethics. All MoBa participants provided written informed consent before enrolment into the study. The MoBa cohort is now based on regulations related to the Norwegian Health Registry Act. The current study was approved by The Regional Committees for Medical and Health Research Ethics (2019/339).

### 2.10. Statistics

We used continuous scores for NND adherence as well as categorized NND scores divided into three categories defined as low, medium, and high adherence (Table 1).

For the outcome measures, scoring of each item in the respective instruments was summarized to total scores for each individual, and a mean value was calculated for each tool. For the screening tools SCARED and Mood and Feelings, cut-off for higher symptomology was set at >1 SD above mean (since >1.5 SD would yield 0 cases). For the NHiPIC-30 continuous variables were used.

Linear models were fitted for each outcome with the age specific NND scores, i.e., prenatal, 6 and 18 months, and 3 and 7 years. Logistic regression models were similarly used to calculate crude and adjusted odds ratios (OR) for higher level of depression or anxiety symptoms with low and medium vs. high NND category.

Analyses were performed in SPSS version 23.0 and STATA/SE 16.0.

## 3. Results

Maternal characteristics are presented according to low, medium and high NND scoring, respectively (Table S3). High NND score is characterized by higher maternal age, lower maternal obesity, higher education, being multiparous and smoking less. There were no differences in breastfeeding and civil status between the different NND groups.

The proportion of participants categorized to low, medium, and high NND score at each assessment are presented in Table 1. The score range varies between assessment points due to the number of subscales in each score, as do the proportion of participants in each of the three categories.

Outcome data at 8 years shows that about 10% of the participants scored high on anxiety symptoms, while 11.7% scored high on depression symptoms (SMFQ) (Table 2).

Table 3 presents score distribution for the five personality traits in the respective dimensions. The mean score is lower for neuroticism, than the other four personality traits (Table 3).

In adjusted analyses we observed an inverse continuous association between NND scoring at 3 and 7 years and the anxiety score at 8 years. A similar association was observed between NND scoring at 3 and 7 years and anxiety and depression symptoms scores at 8 years (Table 4). There was also an association between maternal NND adherence and child depression symptoms at 8 years.

In Table 5 we present associations of anxiety and depression scores with low and medium vs. high NND score. Low vs. high NND score at 3 and 7 years was associated with higher odds of anxiety (OR: 1.1 (95% CI 1.0, 1.3) and OR: 1.2 (95% CI 1.1, 1.3), respectively). Low vs. high NND score at 7 years was slightly associated with depressive symptoms at 8 years (OR: 1.0 (95% CI 1.0, 1.2)).

Table 6 describes the linear association between continuous age-specific NND scores and each of the five personality traits. All personality scores are scored to reflect higher degree of the personality trait, e.g., higher score on extraversion, means being more extraverted. We observed positive associations between NND score and extraversion at all timepoints assessed, except at 6 months where higher NND score was associated with lower score on extraversion. There was a positive association between NND scores and benevolence at all time points, except at 6 months. An inverse association between maternal diet and child diet at 3 and 7 years and neuroticism, was observed. The higher NND score the lower score on neuroticism. There was a positive association between NND scores and conscientiousness at all time points except at 6 months. There was also a positive association between NND scores and imagination at all time points.

## 4. Discussion

The complex processes of early growth and development in infants have been defined as the most important targets of nutrition research, yet poorly studied [40]. As one of the first, we present robust associations between diet early in life and the big five personality traits reported by parents at child aged 8 years using data from the large Norwegian Mother, Father and Child cohort. We also report on associations between early diet and symptoms of anxiety and depression in later childhood with indications that a less healthy and sustainable diet in early life is predictive of more psychiatric symptoms.

Our results indicate that maternal adherence to the NND during pregnancy is associated with parents reporting lower scoring on the depression scale when the child is 8 years old, however, no significant association with anxiety. Jacka et al. (2013) reported from the same cohort that maternal diet during pregnancy was independently related to internalizing and externalizing behavior in children at 5 years of age [11], assessed with the Child Behaviour Checklist that captures similar constructs as the NHiPIC-30 [36]. Borge et al. (2021) found that maternal overall diet quality during pregnancy was associated with a small decrease in offspring ADHD symptom score at 8 years in MoBa and lower risk of ADHD diagnosis [12]. Further, in Project Viva, Mahmassani et al. (2022) found maternal diet quality during pregnancy to be associated with better spatial skills at early childhood, and better verbal intelligence and executive function in the offspring at mid-childhood [14]. In addition, Mortaji et al. (2021) showed that healthier maternal pregnancy diet could be linked to better child neurodevelopment in families living in suboptimal environments [13].

We also investigated whether early child diet, at 6 months, 18 months, 3 and 7 years were associated with symptoms of anxiety and depression at 8 years. Interestingly we find more robust association of anxiety and depression with diet at age 3 and 7 years compared to earlier ages. Jacka et al. [11] found that lower intake of healthy foods in early childhood was associated with higher levels of both internalizing and externalizing problems at five years, while Borge et al. found no association with early postnatal diet quality and later ADHD (at 8 years) [12]. We have previously reported cross-sectional associations between diet at one year of age and measures of neurodevelopment [41]. In total, these findings combined underpin a probable impact of both maternal pregnancy diet and early childhood diet on several aspects of child mental health.

Interestingly, no studies have so far reported on associations between early life diet and the five big personality traits. However, indirectly other studies reporting on diet in relation to outcomes such as depression, anxiety, internalizing and externalizing behavior probably captures parts of the same constructs [11,12]. We find that a healthy and sustainable maternal diet during pregnancy is associated with higher trait scores on extraversion, benevolence, conscientiousness, and imagination, while it yields lower scores on neuroticism. The same relation holds for child diet, with the most robust relations with the various traits at 8 years seen with diet score at 3 and 7 years. Albeit the scarcity of literature on the big five and association to diet, we have identified two studies where maternal diet is linked to child temperament, showing that healthy diet is related to easier temperament [18,20].

The observed associations of NND scoring at different ages up to seven years with aspects of mental health and personality traits at 8 years may be underpinned by differential mechanisms due to the child’s developmental stage at the time of measurement. Associations between early life diet (maternal and child) and child symptoms of depression and anxiety may work through direct pathways such as availability of nutrients and antioxidants in the circulation during critical phases of rapid brain growth development [7]. During fetal life it may also work through indirect pathways related to the prevention of pregnancy complications that affect nutritional flow across placenta and fuel allocation in the fetus [42]. We have previously shown that high vs. low maternal NND score in mid-pregnancy was associated with higher birthweight (direct) and lower risk of preeclampsia and preterm delivery (indirect) in the MoBa cohort [43]. Maternal diet and nutritional health during pregnancy may also work through epigenetic modifications that confer stable patterns of gene expression [44].

What does higher NND-scoring imply nutritionally at the various time points that could explain the observed associations with mental health symptoms and personality traits at 8 years of age? Fruits, vegetables, potatoes, fish, milk, and more drinking water vs. sweetened beverages are included in all scores. Most scores also have whole grains included (except the one applied at 3 years). Combined, these foods are important components of a balanced diet supplying essential micronutrients in addition to dietary fibre, antioxidants and long chain fatty acids. The 6-month diet score differ from the other scores by mainly reflecting breastfeeding exposure (exclusivity and duration) and the degree to which the infant receives homemade relative to commercial baby foods. We speculate that the inverse association with extraversion only observed at 6 months could be related to the symbiotic relationship with the mother in the first months of life.

### Strengths and Limitations

This study has several strengths. To our knowledge, associations between early diet and personality traits have not been presented previously. We used data from a well-described, large, prospective population-based birth cohort with possibility to adjust for a broad range of potential confounders. The instruments used to assess mental health and personality traits are valid and reliable.

There are, however, limitations that should be addressed. All variables used in the analyses were self-reported by the mother. Mothers participating in MoBa were older, cohabitating, non-smokers and frequent users of multivitamins and folic acid supplements [45] and thus not fully representative of the background population. Measures of association are, however, unlikely to be biased as shown in previous MoBa analyses [45].

The age-specific NND scores differ somewhat in composition, leading to slight differences in the dietary and nutritional composition they reflect. As the questions regarding child diet were less detailed than in the maternal questionnaire, neither exact amount eaten, nor nutrient intake could be calculated. We do not know whether and to which degree energy and nutrient intake varied across scoring in the respective child NND scores. This is especially true for the NND score at 6 months where the score variability was mainly related to breastfeeding vs. formula and homemade vs. commercial baby foods.

Are the observed associations with personality traits likely to be causal? The effect sizes for the adjusted associations between diet scores and both conscientiousness and imagination were quite stable when assessed across time and larger than for the other traits. Some associations could be subject to reciprocal mechanisms, meaning that stronger personality traits are associated with food acceptance, dietary choices, likes and dislikes [46]. The fact that maternal NND score is independently associated with personality at 8 years lend support to the idea that diet/nutritional status in itself may influence personality development.

We did not correct for multiple testing. The findings should be considered explorative and be interpreted with caution.

## 5. Conclusions

Our results indicate that healthy diet early in life may impact mental health and personality development during childhood. If adherence to a healthy and sustainable diet influences mental health, this will be important in a public health perspective even if effect sizes are small. It is important to note that our data does not imply causality, and findings should not be overemphasized before replicated in other data sets.

## Figures and Tables

**Table 1:** Detailed description of the different New Nordic Diet (NND) scores (exposure) at time of assessment. Valid proportion of participants in each NND category.

New Nordic Diet Score	N	Mean NND Score (SD)	Score Range	Median	Low Score %	Medium Score %	High Score %
Maternal pregnancy diet	40,566	5.0 (2.0)	0–10	5	24.6	34.8	40.6
Child diet 6 months	39,138	3.4 (1.3)	0–6	3	24.9	55.5	19.6
Child diet 18 months	36,865	4.3 (1.7)	0–9	4	32.5	43.5	24.0
Child diet 3 years	33,464	2.8 (1.4)	0–6	3	19.1	49.4	31.5
Child diet 7 years	34,588	4.7 (1.7)	0–9	5	24.7	43.5	31.8

**Table 2:** Number of participants in the analyses and child mental health outcomes at 8 years. The symptoms scales are ranged from low score reflecting few symptoms and high score reflecting more symptoms.

Mental Health	N	Mean (SD)	Median	Score Range	High Score * N (%)
Anxiety ^1^	40,151	6.0 (1.2)	6	5–15	4119 (10.3)
Depression ^2^	39,532	14.9 (2.4)	14	13–39	4607 (11.7)

^1^ The Screen for child anxiety-related disorders (SCARED) ^2^ The Short Mood and Feelings Questionnaire (SMFQ) * High score was set at ≥1 SD above mean.

**Table 3:** Child personality traits ^1^. Higher scores indicate higher degree of the specific trait.

Child Personality Traits ^1^	N	Mean (SD)	Median	Range (Min–Max)
Extraversion	40,055	21.9 (3.5)	22	5–30
Benevolence	39,780	21.5 (3.7)	22	6–30
Neuroticism	39,998	14.2 (3.2)	14	5–29
Conscientiousness	39,984	21.9 (3.6)	22	6–30
Imagination	39,987	23.6 (3.3)	24	6–30

^1^ The short form of Hierarchical Personality Inventory for children (NHiPIC-30).

**Table 4:** Associations between NND as continuous score and the anxiety and depression symptom scores, respectively. Crude and adjusted ^1^ linear regression. The symptoms scales are ranged from low to higher degree of symptoms.

NND Score		Anxiety Symptoms ^2^	Depression Symptoms ^3^
		β (95% CI)	β (95% CI)
Maternal score	Crude	−0.00 (−0.01, 0.00)	−0.04 (−0.05, −0.03) *
	Adjusted	−0.00 (−0.01, 0.00)	−0.02 (−0.03, −0.01) *
6 months	Crude	0.01 (−0.0, 0.01)	−0.04 (−0.06, −0.02) *
	Adjusted	0.01 (−0.01, 0.02)	−0.00 (−0.02, 0.02)
18 months	Crude	−0.00 (−0.01, 0.01)	−0.03 (−0.04, −0.01) *
	Adjusted	−0.00 (−0.01, 0.01)	−0.01 (−0.02, 0.01)
3 years	Crude	−0.01 (−0.02, −0.01) *	−0.05 (−0.07, −0.03) *
	Adjusted	−0.01 (−0.02, −0.01) *	−0.03 (−0.05, −0.01) *
7 years	Crude	−0.02 (−0.03, −0.01) *	−0.04 (−0.06, −0.03) *
	Adjusted	−0.02 (−0.03, −0.01) *	−0.03 (−0.05, −0.02) *

^1^ Adjusted for maternal age, civil status, maternal education, maternal BMI, maternal depression, parity, siblings included the study population, child age at completion of questionnaire, gestational age in analysis of child diet. ^2^ The Screen for child anxiety-related disorders (SCARED) ^3^ The Short Mood and Feelings Questionnaire (SMFQ). * *p*-value < 0.05.

**Table 5:** Associations between categories of NND as total score and higher levels of anxiety and depression symptoms. Logistic regression (Crude and Adjusted ^1^).

NND		Anxiety Symptoms ^2^			Depression Symptoms ^3^		
		Low	Medium	High	Low	Medium	High
maternal	Crude	1.0 (0.9, 1.1)	1.0 (0.9, 1.1)	1	1.2 (1.1, 1.2) *	1.1 (1.0, 1.2) *	1
	Adjusted	1.0 (0.9, 1.1)	1.0 (0.9, 1.1)	1	1.0 (0.9, 1.1)	1.0 (0.9, 1.1)	1
6 months	Crude	1.0 (0.9, 1.1)	1.0 (0.9, 1.1)	1	1.2 (1.1, 1.3) *	1.0 (0.9, 1.1)	1
	Adjusted	0.9 (0.9, 1.1)	1.0 (0.9, 1.1)	1	1.0 (0.9, 1.1)	1.0 (0.9, 1.1)	1
18 months	Crude	1.0 (0.9, 1.1)	1.0 (0.9, 1.1)	1	1.1 (1.0, 1.2) *	1.0 (0.9, 1.1)	1
	Adjusted	1.1 (0.9, 1.1)	1.0 (0.9, 1.1)	1	1.0 (0.9, 1.1)	0.9 (0.9, 1.1)	1
3 years	Crude	1.2 (1.0, 1.3) *	1.1 (0.9, 1.2)	1	1.2 (1.1, 1.3) *	1.1 (1.0, 1.2) *	1
	Adjusted	1.1 (1.0, 1.3) *	1.0 (0.9, 1.1)	1	1.0 (0.9, 1.2)	1.0 (0.9, 1.1)	1
7 years	Crude	1.2 (1.1, 1.3) *	1.0 (0.9, 1.1)	1	1.2 (1.1, 1.3) *	1.1 (1.0, 1.2) *	1
	Adjusted	1.2 (1.1, 1.3) *	1.0 (0.9, 1.1)	1	1.0 (1.0, 1.2) *	1.0 (0.9, 1.1)	1

^1^ Adjusted for maternal age, civil status, maternal education, maternal BMI, maternal depression, parity, siblings included the study population, child age at completion of questionnaire, gestational age in analysis of child diet. ^2^ The Screen for child anxiety-related disorders (SCARED) ^3^ The Short Mood and Feelings Questionnaire (SMFQ) High score was set at ≥1 SD above mean in both instruments * *p*-value < 0.05.

**Table 6:** Linear regression association between NND scores and child Personality traits ^1^ (Crude and adjusted ^2^).

Child Personality Trait		Extraversion	Benevolence	Neuroticism	Conscientiousness	Imagination
NND Score		β (95% CI)	β (95% CI)	β (95% CI)	β (95% CI)	β (95% CI)
Maternal score	Crude	0.07 (0.05, 0.08) *	0.05 (0.03, 0.07) *	−0.06 (−0.08, −0.05) *	0.12 (0.11, 0.14) *	0.14 (0.12, 0.16) *
	Adjusted	0.06 (0.04, 0.08) *	0.05 (0.03, 0.07) *	−0.02 (−0.04, −0.01) *	0.10 (0.08, 0.12) *	0.12 (0.11, 0.14) *
6 months	Crude	−0.10 (−0.12, −0.07) *	0.01 (−0.02, 0.04)	0.02 (−0.01, 0.05)	0.04 (0.01, 0.06) *	0.13 (0.10, 0.15) *
	Adjusted	−0.06 (−0.09, −0.04) *	−0.00 (−0.03, 0.03)	0.02 (−0.00, 0.04)	0.01 (−0.02, 0.04)	0.09 (0.06, 0.12) *
18 months	Crude	0.05 (0.03, 0.08) *	0.08 (0.06, 0.10) *	−0.02 (−0.04, −0.00) *	0.11 (0.08, 0.13) *	0.20 (0.18, 0.22) *
	Adjusted	0.07 (0.04, 0.09) *	0.07 (0.05, 0.10) *	−0.02 (−0.04, 0.00)	0.09 (0.07, 0.11) *	0.18 (0.16, 0.20) *
3 years	Crude	0.09 (0.06, 0.11) *	0.14 (0.11, 0.17) *	−0.10 (−0.12, −0.07) *	0.18 (0.15, 0.21) *	0.24 (0.21, 0.26) *
	Adjusted	0.11 (0.08, 0.14) *	0.13 (0.10, 0.16) *	−0.09 (−0.12, −0.07) *	0.16 (0.13, 0.19) *	0.21 (0.19, 0.24) *
7 years	Crude	0.04 (0.02, 0.06) *	0.14 (0.11, 0.16) *	−0.04 (−0.05, −0.02) *	0.14 (0.12, 0.17) *	0.18 (0.16, 0.20) *
	Adjusted	0.06 (0.04, 0.09) *	0.14 (0.11, 0.16) *	−0.06 (−0.08, −0.04) *	0.14 (0.12, 0.16) *	0.16 (0.14, 0.18) *

^1^ Child personality traits measured by the short form of Hierarchical Personality Inventory for children (NHiPIC-30) ^2^ Adjusted for maternal age (years) at delivery, parity, child sex, and maternal education level, marital status, energy intake (KCAL), maternal depression, and pre-pregnancy BMI. * *p*-value < 0.05.

## Data Availability

Data from the MoBa study need to be applied for from the Norwegian Institute of Public Health.

## Supplementary Materials

The following supporting information can be downloaded at: https://www.mdpi.com/article/10.3390/nu15010243/s1; Table S1: Overview of the diet scores and the corresponding subscales assessing adherence to a potentially healthy and sustainable diet (NND) during pregnancy and child age 6 months, 18 months, 3 years and 7 years. Table S2: The Norwegian Short Form of the Hierarchical Personality Inventory for Children. Scale of 5 choices (1—Not typical, 2—Not very typical, 3—Quite typical, 4—Typical, 5—Very typical). Table S3: Maternal characteristics according to NND adherence during pregnancy for participants answering the 8 year questionnaire.
