# Supplementary material for: Diet in Early Life Is Related to Child Mental Health and Personality at 8 Years: Findings from the Norwegian Mother, Father and Child Cohort Study (MoBa)
Source: Nutrients. 2023 Jan 3;15(1):243. doi: 10.3390/nu15010243 (PMC9823869; doi:10.3390/nu15010243)
Supplement: Supplementary file 1 [file nutrients-15-00243-s001.zip › nutrients-2089545-supplementary.pdf]

**Table S1:** Overview of the diet scores and the corresponding subscales assessing adherence to a potentially healthy and sustainable diet (NND) during pregnancy and child age 6 months, 18 months, 3 years and 7 years.

| Maternal score | 6-months score | 18-months score | 3-years score | 7-years score |
|---|---|---|---|---|
| **Scoring range:** 0-10<br>**Categories:** Low 0-3, medium 4-5, high 6-10 | **Scoring range:** 0-6<br>**Categories:** Low 0-2, medium 3-4, high 5-6 | **Scoring range:** 0-9<br>**Categories:** Low 0-3, medium 4-5, high 6-9 | **Scoring range:** 0-6<br>**Categories:** Low 0-1, medium 2-3, high 4-6 | **Scoring range:** 0-9<br>**Categories:** Low 0-3, medium 4-5, high 6-9 |
| **1) Meal pattern:**<br>breakfast, lunch, dinner and evening meal. | **1)** Consuming more **HM fruit puree** relative to CP fruit puree | **1) Fruits:**<br>eating fruits more than 10.5 t/week | **1) Fruits:**<br>eating fruits more than 7 t/week | **1) Local fruits:**<br>eating apple, pear and grapes more than 3.5 t/week |
| **2) Nordic fruits:**<br>apples, pears, plums and strawberries. | **2)** Consuming more **HM dinners** relative to CP dinners | **2) Vegetables:**<br>eating vegetables more than 5.5 t/week | **2) Vegetables:**<br>eating vegetables more than 5 t/week | **2) Root vegetables:**<br>eating carrots more than 1.5 t/week |
| **3) Root vegetables:**<br>carrots, rutabaga and various types of onions. | **3)** Consuming more **HM porridge** over CP porridge | **3) Peas and beans:**<br>eating peas and beans more than 5 t/week | **3) Potatoes:**<br>eating more potatoes over rice and pasta. | **3) Cabbages:**<br>eating kale, cauliflower and broccoli more than 1.5 t/week |
| **4) Cabbages:**<br>kale, cauliflower, broccoli and Brussels sprouts. | **4)** Being exclusively breast-fed for at least 4 months (**yes**/no) | **4) Potatoes:**<br>eating more potatoes over rice and pasta. | **4) Fish:**<br>eating fish more than 2.12 t/week | **4) Potatoes:**<br>eating more potatoes over rice and pasta. |
| **5) Potatoes:**<br>eating more potatoes over rice and pasta | **5)** Any breastfeeding at 6 months (**yes**/no) | **5)** Eating more **HM porridge/baby cereal** over CP porridge/baby cereal | **5) Milk:**<br>drinking more milk over fruit juice | **5) Whole grain bread:**<br>reporting no consumption of white bread |
| **6) Whole grain breads:**<br>eating more whole grain breads over refined breads | **6)** Drinking more **water** over sweetened beverages | **6) Fish:**<br>eating fish more than 2.13 t/week | **6) Sweet beverages:**<br>drinking sweetened beverages less than 2.5 t/week | **6) Oatmeal:**<br>eating muesli or oatmeal more than 1.5 t/week |
| **7) Oatmeal porridge:**<br>frequency of eating oatmeal porridge | | **7) Milk:**<br>drinking more milk over fruit juice | | **7) Fish:**<br>eating fish more than twice a week |
| **8) Foods from the wild countryside:**<br>game, fish, seafood and native berries. | | **8) Water:**<br>drinking more water over sweetened beverages | | **8) Milk:**<br>drinking more milk over fruit juice |
| **9) Milk:**<br>drinking more milk over fruit juice | | **9)** Eating more **HM dinners** over CP baby food | | **9) Water:**<br>drinking more water over sweetened beverages |
| **10) Water:**<br>drinking more water over sweetened beverages | | | | |

Abbreviations: HM= homemade, CP= commercially prepared, t/week = times a week.

**Table S2.** The Norwegian Short Form of the Hierarchical Personality Inventory for Children. Scale of 5 choices (1-Not typical, 2- Not very typical, 3- Quite typical, 4 – Typical, 5- Very typical)

| **The Big Five personality Factors** | Question no and text from MoBa 8y questionnaire |
|---|---|
| **Extraversion** | |
| | 8.Has energy to spare |
| | 10.Talks to people easily |
| | 18. Is chatty |
| | 19. Enjoys life |
| | 23. Has an infectious laugh |
| | 25. Talks about own feelings |
| **Benevolence** | |
| | 4. Obeys without protests |
| | 5. Takes himself/herself into consideration first* |
| | 11. Does everything to get his/her own way* |
| | 16. Imposes her or his will* |
| | 21. Is easily incensed by things* |
| | 28. Doesn't envy others |
| **Neuroticism** | |
| | 1.Is easily caught up in problems |
| | 6. Is quick to worry about things |
| | 14. Doubt himself/herself |
| | 17. Is readily discouraged by imminent failure |
| | 22. Is quick to doubt his/her own capacities |
| | 27. Has confidence in own abilities* |
| **Conscientiousness** | |
| | 3. Makes an all-out effort |
| | 7. Forgets anything and everything* |
| | 9. Prefers to leave work to others* |
| | 13. Is not very thorough* |
| | 15. Finishes tasks to the very end |
| | 26. Carries out work to the last detail |
| **Imagination** | |
| | 2. Has a broad range of interests |
| | 12. Derives pleasure from creating things |
| | 24. Has a rich imagination |
| | 20. Is quick to understands things |
| | 29. Is interested in all that is new (is interested in anything) |
| | 30. Can express himself/herself well |

*Indicate that in this question the original scale has been reversed to comply with the total scale.

**Table S3**. Maternal characteristics according to NND adherence during pregnancy for participants answering the 8 year questionnaire.

| | Study population | Maternal pregnancy NND score | | |
|---|---|---|---|---|
| Maternal age at delivery | n(%) | Low n(%) | Medium n(%) | High n(%) |
| <25y | 3,189(7.9) | 1,160(11.6) | 1,134(8.0) | 895(5.4) |
| 25-29y | 13,004(32.1) | 3,623(36.3) | 4,634(32.8) | 4,747(28.9) |
| 30-34y | 16,590(40.9) | 3,748(37.5) | 5,821(41.2) | 7,021(42.7) |
| 35y+ | 7,783(19.1) | 1,460(14.6) | 2,535(18.0) | 3,788(23.0) |
| Prepregnancy BMI | | | | |
| <18,5 | 1,099(2.7) | 291(2.9) | 394(2.8) | 414(2.5) |
| 18,5-24,9 | 26,838(66.2) | 6,182(61.9) | 9,340(66.1) | 11,316(68.8) |
| 25-29,9 | 8,457(20.9) | 2.304(23.1) | 2,881(20.4) | 3,272(19.9) |
| 30+ | 3,298(8.1) | 985(9.8) | 1,229(8.7) | 1,084(6.6) |
| Missing | 874(2.1) | 229(2.3) | 280(2.0) | 365(2.2) |
| Maternal education | | | | |
| =<12 y | 9,828(24.2) | 2,922(29.3) | 3,455(24.5) | 3,451(21.0) |
| 13-16 y | 18,262(45.0) | 4,394(44.0) | 6,362(45.0) | 7,506(45.6) |
| 17+ y | 11,722(28.9) | 2,479(24.7) | 4,031(28.5) | 5,221(31.7) |
| missing | 754(1.9) | 205(2.0) | 276(2.0) | 273(1.7) |
| Parity | | | | |
| primiparous | 18,543(45.7) | 5,230(52.4) | 6,759(47.9) | 6,554(39.8) |
| multiparous | 22,023(54.3) | 4,761(47.6) | 7,365(52.1) | 9,897(60.2) |
| Smoking during pregnancy | | | | |
| no smoking | 38,082(93.9) | 9,202(92.1) | 13,240(93.7) | 15,640(95.1) |
| smoking | 2,244(5.5) | 730(7.3) | 812(5.8) | 702(4.3) |
| missing | 240(0.6) | 59(0.6) | 72(0.5) | 109(0.6) |
| Breastfeeding | | | | |
| yes | 38,747(95.5) | 9,499(95.1) | 13,499(95.6) | 15,749(95.7) |
| no | 1,819(4.5) | 492(4.9) | 625(4.4) | 702(4.3) |
| Civil status married/cohabitant | | | | |
| yes | 39,379(97.1) | 9,646(96.6) | 13,708(97.1) | 16,025(97.4) |
| no | 1,007(2.5) | 302(3.0) | 358(2.5) | 347(2.1) |
| missing | 180 (80.4) | 43(0.4) | 48(0.4) | 79(0.5) |
